# Bilirubin 2022: The Past, the Present and the Future

**DOI:** 10.3390/antiox11091632

**Published:** 2022-08-23

**Authors:** Cristina Bellarosa, Claudio Tiribelli

**Affiliations:** Italian Liver Fondation, Bldg. Q—AREA Science Park Basovizza, SS14, km. 163,5, 34149 Trieste, Italy

The present Special Issue (SI) addresses the double-faced Janus behavior of bilirubin. Bilirubin becomes neurotoxic if serum concentrations are severely elevated, protective when mildly increased, and, if below the accepted lower reference value of 0.3 mg/dL or 5 µmol/L, it correlates with a higher risk of cardiovascular and metabolic diseases

The global bilirubin network that contributed to the SI covered both the protective and neurotoxic sides of the coin demonstrating that the interest in the yellow pigment involves long-standing researchers from the USA, Europe, Australia, and Asia.

The most studied aspect is the neurotoxic effect that the pigment could provoke in severely jaundiced neonates causing long-life neurological damage. Despite an increased comprehension of the molecular mechanisms involved in bilirubin neurotoxicity, the golden standard method for the treatment remains phototherapy. Opportunely Capkova et al. [1] evaluate the side toxic effects of bilirubin photo derivatives on human neurodevelopment, while Bortolussi et al. [2] propose a novel strategy to inhibit bilirubin production and prevent neurotoxicity in mice. Based on the rescue effect of genetic inactivation of Biliverdin Reductase a (BVRA) on a lethal mice model of severe Hyperbilirubinemia, they propose the idea that pharmacological inhibition of BVRA could prevent irreversible neurological damage in severely hyperbilirubinemic neonates.

On the protective side, many scientists support the use of serum bilirubin levels as predictors of cardiovascular diseases (CVD), obesity, and type 2 diabetes. Ho et al. [3] propose an accurate risk stratification for CDV in hemodialysis patients according to polymorphism of the enzymes that contribute to elevated serum bilirubin. The main features of the so-called “civilization’s disease” (obesity, type 2 diabetes, CVD) are oxidative stress and inflammation. Chronic mild unconjugated hyperbilirubinemia occurring in Gilbert subjects correlates with increased antioxidant status and reduced pro-oxidant and pro-inflammatory markers [4]. Obesity and Type 2- diabetes are risk factors for non-alcoholic fatty liver disease (NAFLD), but the relationship between circulating total bilirubin and the incidence of NAFLD is uncertain. The polymorphism affecting bilirubin metabolism does not have any effect on NAFLD development, while the metabolism of bilirubin seems to be dysregulated due to the increased oxidative stress caused by liver damage [5].

Considering the benefit for human health coming from chronic mild unconjugated hyperbilirubinemia as in Gilbert subjects, it is suggested that iatrogenically mimicking Gilbert Syndrome in the entire population or at least in the disease group as overweight and obese adults [6]. Natural compounds or synthetic drugs able to modulate the enzymes involved in bilirubin synthesis are already available, but the clinical and ethical feasibility of this approach should be verified. In the last five years, novel approaches to using bilirubin as a pharmacological agent were developed. Recently the nanoparticle encapsulation and the synthesis of bilirubin analog allow for overcoming the problem of synthesizing this chemically complicated and water-insoluble molecule and open the door to the possibility to deliver bilirubin where its anti-inflammatory and antioxidant effects are needed [7].

From being considered a non-functional waste product, a sign of liver disease, or only as a potentially neurotoxic substance, our understanding of bilirubin has come a very long way, but future research is certainly needed. The present SI takes a step forward to better investigate the many intricate biological functions of this molecule, which has been recently recognized as a hormone.
